# Effect of Compaction Ratio on Mechanical Properties of Low-Strength Hydraulically Bound Mixtures for Road Engineering

**DOI:** 10.3390/ma15041561

**Published:** 2022-02-19

**Authors:** Cezary Kraszewski, Leszek Rafalski, Beata Gajewska

**Affiliations:** 1 Road and Bridge Research Institute IBDiM, Instytutowa 1, 03 302 Warsaw, Poland; lrafalski@rgib.org.pl; 2 Department of Geotechnical Engineering, Institute of Civil Engineering, Warsaw University of Life Sciences, Nowoursynowska 159, 02 776 Warsaw, Poland; beata_gajewska@sggw.edu.pl

**Keywords:** compaction ratio, soil stabilisation, capping layer, compacting energy, road engineering, cement–soil

## Abstract

Road layers should be properly compacted to obtain an adequate bearing capacity and durability. Both the unbound and hydraulically bound mixtures used in the layers require compaction. After compaction and hardening, soil mixed with a binder acquires mechanical features that unbound soil lacks, including tensile strength (*R_it_*) and unconfined compressive strength (*R_c_*). The effect of the compaction ratio (*D_Pr_*) of the low-strength cement-stabilised soils on these features has rarely been investigated. This study investigates the influence of the compaction ratio on the mechanical properties of hardened, stabilised mixtures of medium-grained sand with 5%, 6.5%, and 8% Portland cement. Cement–soil stabilisation tests showed that compressive strength depends exponentially on the compaction ratio, whereas tensile strength and the stiffness modulus depend linearly on the compaction ratio. For tensile strength and the dynamic stiffness modulus, the effect is not statistically significant, and the usual practice of ignoring compaction dependence is justified. For compressive strength, however, the effect is significant, especially when *D_Pr_* = 98–100%. When the values of *R_c_* and *R_it_* strengths at various *D_Pr_* were normalised by those at 100%, it was found that mixtures with higher strengths are the least resistant to changes in the compaction ratio. Knowing the percentage by which the value of a given parameter changes with compaction can be extremely valuable in engineering practice.

## 1. Introduction

Soil stabilisation through the addition of a binder (and water, if needed) in road construction increases or maintains the strength and durability by hydraulic binding or carbonation. This method is widely used for increasing the load-bearing capacity of weak subgrades by making capping layers and for executing road-base layers. After compaction and hardening, the soil–binder mixture acquires mechanical properties unlike those of unbound soil. These properties include indirect tensile strength (*R_it_*) and unconfined compressive strength (*R_c_*), both due to the bonding of the grout to the soil. The mixture of the bonded soil with cement results in a significant increase in the load-bearing capacity and strength of the subgrade. In addition, by transferring tensile forces, the soil layer stabilised by binders allows for a better distribution of loads than in unbound (non-stabilised) soils, and it is more durable and resistant to weather conditions, such as moisture and frost.

The most commonly used soil stabilisers include Portland cement, lime (hydrated or quicklime), fly ash, hydraulic road binders [1,2,3,4,5], chemical stabilisers, polymers, bitumen emulsion [6,7,8], and by-products [9,10]. The soil forms a semi-rigid layer after it is mixed with the binder and undergoes compaction and hardening. This is because its strength parameters have smaller values (*R_c_* < 5 and *R_it_* < 0.5 MPa) compared with those of traditional concretes. However, these low strengths significantly increase the load-bearing capacity of the stabilised subgrade, as determined by deformation modulus (*Ev*) tests conducted in situ. This is beneficial because the stabilised subgrade of road structures has a relatively high elasticity and low susceptibility to shrinkage cracking.

Two systems are used in the design of binder-stabilised road layers: European Standard EN 14227-1 [11] and EN 14227-15 [1]. One is based only on the unconfined compressive strength, *R_c_,* and the other on the indirect tensile strength, *R_it_*, and the modulus of elasticity, *E* (stiffness modulus), after a contractual period of curing [12,13,14] under laboratory conditions. The curing time, which ranges from 28 to 360 days, depends on the type of binder used. Conventionally, 28-day curing is applied to cement-stabilised mixtures. Binder-stabilised soil specimens are normally compacted based on the required compaction ratio. After curing, the aforementioned mechanical properties are determined. This applies to laboratory conditions in which the mixture formula is determined for implementation in the field. 

In practice, low-bearing soils are often stabilised using the in situ method [2,5] in a layer with a thickness of 15–40 cm, which is then compacted with a roller. A subgrade of low bearing capacity may occur under the stabilised layer, making it difficult or even impossible to compact the stabilised layer to the *D_Pr_* values assumed in the mixture design. This assumption is true for silty and clay soils, which become plasticised during the compaction process.

In road engineering, compaction is determined by the compaction ratio, *D_Pr_*:

(1) $$D_{Pr}=\frac{\rho_{d}}{\rho_{ds}}\times 100\%\;$$

where *ρ_d_* (g/cm^3^) is the dry density of the soil skeleton determined in the field by a direct test and *ρ_ds_* (g/cm^3^) is the laboratory maximum dry density of the soil skeleton using the Proctor test based on the European Standard EN 13286-2 [15].

European Standard EN 13286-1 [16] allows the vibration hammer, vibration press, static press, or the Proctor apparatus to be used to determine the maximum dry density of the skeleton of unbound and bound soils and mixtures. Each of these has a different effect on the compaction of the material, and the methods cannot be interchanged [17,18,19]. The consistent use of one selected compaction method throughout the stabilisation mixture design process is required.

The Proctor test is one of the most commonly used methods to simulate real field conditions in a controlled laboratory environment [3,20], and the tests presented in this study were conducted using this method. The required values of the compaction ratio in traffic engineering usually range between 98% and 103% *D_Pr_*, as determined by the Proctor test. Typically, *D_Pr_* ≥ 100%, and the requirements for other material properties after compaction and hardening (e.g., compressive strength, *R_c_*, indirect tensile strength, *R_it_,* California Bearing Ratio, CBR, and deformation modulus, *Ev*) are related to this value [21,22,23].

The mixture of soil and binder forms a load-bearing skeleton after the hardening process. The soil particles that are not bound by the binder act as a filler of the load-bearing skeleton and a shock absorber of external forces, imparting susceptibility [24]. The unbound soil also serves as a shock absorber of internal forces (such as those due to temperature and shrinkage), causing soil stabilised by binders to be characterised by lower shrinkage than cement concretes. It has a completely different structure (semi-rigid) than unbound soil (flexible) or bound concrete (rigid). Although most of the relationships between the mechanical properties of hydraulically bound and unbound soils [25,26] have been established and properly documented, the mechanics of soils stabilised with a small amount of binder have not been studied. Analysed studies have mainly examined the differences in laboratory compaction methods [20,21,27,28] or focused on the effect of binders and different stabilisation additions [1,11,29], as well as the comparison of individual test methods, e.g., tests between the static and dynamic stiffness modulus [30]. Some of the research on soil stabilisation and its compaction concerned mixtures of high strength or the effect of a delayed compaction time and its influence on the parameters of the bound mixture, which is very important in this technology [31,32].

The analysed studies proved the significant influence of compaction on unbound soils due to their deformation properties. Regarding stabilised soils, does this indicate that compaction can be considered less important in the case of slightly stabilised soils with binders due to their lower deformability than unbound soils?

This study examines the justifiability of the above considerations. It presents the results of laboratory tests to determine the effect of compaction conditions on the mechanical properties of low-strength cement-stabilised soils. The individual values of compressive strength and indirect tensile strength were determined according to standard procedures [33,34]. To determine the dynamic stiffness modulus, a standard method [35] was used with the necessary modifications considering the specificity of these mixtures, described further in Section 2.3.3.

A novelty is that the tests were carried out on low-strength stabilisation mixes, using three Portland cement additives, and the samples were compacted with different compaction energies corresponding to 95%, 98%, and 100% *D_Pr_*. Based on the research, the percentage values of the mechanical parameters under different compaction ratios in relation to values at 100% *D_Pr_* were established. Furthermore, the percentage values can be used to predict how weak the strength parameter will be in relation to the assumptions made at 100% *D_Pr_*, which may have engineering utility.

## 2. Materials and Methods

### 2.1. Materials

**Figure 1:**
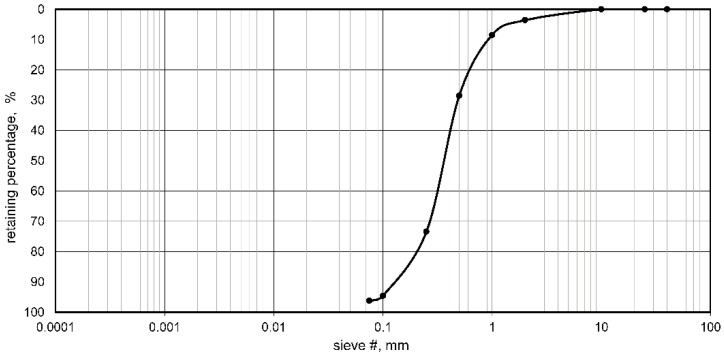
Gradation curve of the sand used in this study.

In this study, Portland cement CEM I 42.5R was used as a binder in compliance with the European Standard EN 197-1 [36]. Medium-grained sand was employed in the tests. The grain size distribution is shown in Figure 1.

### 2.2. Specimen Preparation

The test specimens were cylinders, dynamically compacted to a diameter *ϕ* = 100 mm and height *h* = 120 mm based on the standard Proctor method, with an energy density of 0.59 J/cm^3^. In this study, three levels of specimen compaction were assumed, corresponding to 95%, 98%, and 100% $D_{Pr}$ of the standard max density $D_{Pr}$ determined by the Proctor test. Preliminary tests were conducted to determine the required number of hammer blows per layer (three layers) to achieve the assumed compaction of the specimen. The hammer weighed 2.5 kg and was dropped from a height of 305 mm based on the standard Proctor method, in compliance with the European Standard EN 13286-2 [15].

**Figure 2:**
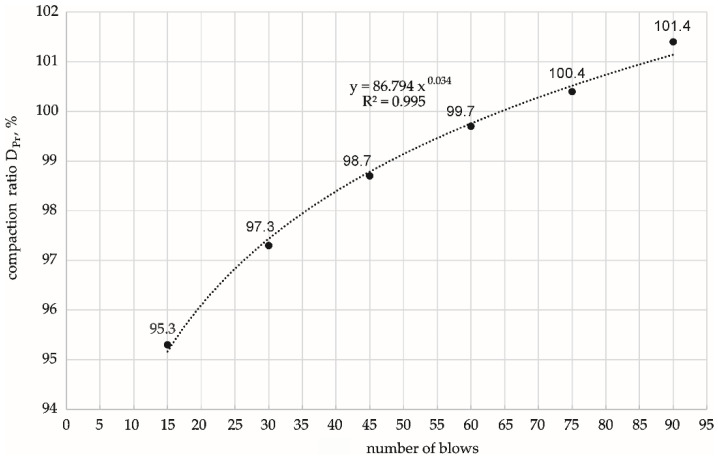
Dependence of the compaction ratio on the total number of hammer blows of the compaction.

Preliminary laboratory tests were conducted to determine the impact quantities and the corresponding compaction ratio, which were found to have an exponential relationship (Figure 2).

Finally, the following numbers of impacts were assumed to compact specimens with a diameter *ϕ* = 100 mm and height *h* = 120 mm into the assumed compaction ratio:

3 × 5 = 15 blows for 95% $D_{Pr}$
3 × 12 = 36 blows for 98% $D_{Pr}$
3 × 25 = 75 blows for 100% $D_{Pr}$

The compaction energy per unit volume of the specimen (E), and the percentage of this energy relative to the standard Proctor energy depending on the number of impacts applied, are summarised in Table 1.

The specimens were dynamically compacted at the optimum moisture content (OMC) specified for each mixture of sand with Portland cement, as shown in Table 2. The actual compaction of the specimens after determining the dry density, $\rho_{d}$ (established on 2 samples of each mixture), was close to the assumed value in the design (i.e., within $D_{Pr}\pm 1\%$) with standard deviation, SD = 0.00–0.0212%$\;D_{Pr}$. A higher $D_{Pr}$ means a higher material density, $\rho_{d}$, implying the closer packing of soil grains and lower pore content.

**Figure 3:**
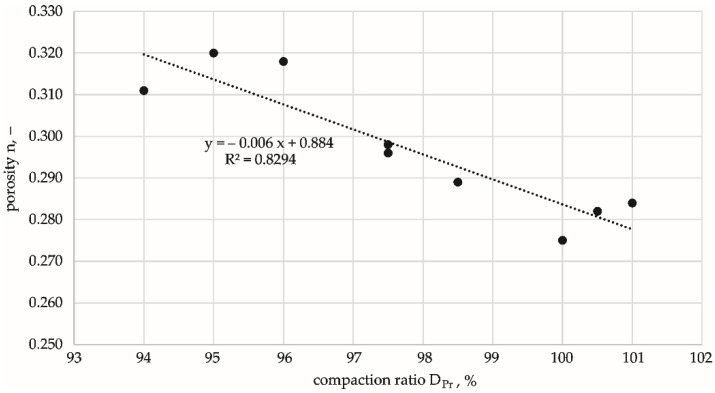
Actual compaction of test specimens vs. porosity.

Figure 3 shows the relationship between the actual compaction of the specimens and the calculated porosity.

(2) $$n=1-\frac{\rho_{d}}{\rho_{s}},$$

where $\rho_{d}$ (g/cm^3^) is the dry density of the soil skeleton and $\rho_{s}$ (g/cm^3^) is the specific density (2.65 g/cm^3^).

The graph shows that the obtained densities of the specimens were within the limits of $D_{Pr}\pm 1\%.\;$ The corresponding porosities (n) were $D_{Pr}$ = 95% and n = 0.315, $D_{Pr}$ = 98% and n = 0.297, and $D_{Pr}$ = 100% and n = 0.283.

The effect of the strength range of the hardened mixture on these relationships and trends can be determined using mixtures with various cement additions. A total of nine mixtures were prepared with three different cement contents (i.e., 5%, 6.5%, and 8% by weight relative to dry soil mass) and three different densities (95%, 98%, and 100% $D_{Pr}$).

The influence of cement addition on the porosity of the tested mixtures is presented below.

**Figure 4:**
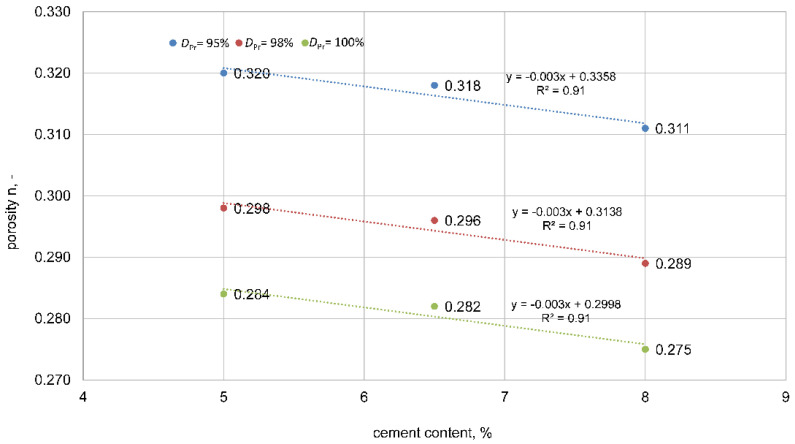
Influence of the addition of cement on porosity. **━** *D*_Pr_ = 95%, **━** *D*_Pr_ = 98%, **━** *D*_Pr_ = 100%.

The dependencies show that the addition of cement is clearly related to the porosity of the mixture. The greater the cement addition, the lower the observed porosity, which should be related to the better filling of the pores with cement in the mixture. The graph confirms the inversely proportional relationship between porosity and the state of compaction, as presented in Figure 4.

The mixtures were designed such that the sum of the masses of all dry components (soil/cement) in the mixture was 100% based on the European Standard EN 14227-15 [1]. The properties of fresh sand and cement mixtures are presented in Table 2.

The specimens were subjected to the typical 28-day curing used for cement-stabilised soils after forming. For 14 days, specimens were stored in an environment with ≥ 95% humidity at 20 ± 2 °C, and another 14 days where the specimens were fully immersed in water at 20 ± 2 °C [3,23]. After 28 days of curing, the compressive strength, indirect tensile strength, and dynamic stiffness modulus of the specimens were determined. The compressive strength tests were performed on cylindrical specimens with *ϕ* = 100 mm and *h* = 120 mm. On the other hand, tensile strength and dynamic stiffness modulus tests were conducted using the indirect tensile method on cylindrical specimens with the same diameter, but *h* = 60 mm. Therefore, specimens with a height *h* = 120 mm were cut in half to allow the use of specimens with identical densities.

### 2.3. Research Programme

Nine combinations of sand mixtures with different cement contents and densities were prepared as described above. The test programme was used to determine the unconfined compressive strength (based on EN 13286-41 [33]), indirect tensile strength (based on EN 13286-42 [34]), and dynamic stiffness modulus (based on EN 12697-26 [35] and previous studies [26,37,38,39]).

Tests were conducted on 3 specimens for each type of test and prepared mixture, yielding a total of 81 specimens. The results of the study were subjected to analysis of variance (ANOVA) at a statistical significance level of α = 0.05.

#### 2.3.1. Unconfined Compressive Strength ($R_{c}$)

Compressive strength tests were conducted on cylindrical specimens with diameter *ϕ* = 100 mm and height *h* *=* 120 mm, based on EN-13286-41 [33]. These specimens were continuously and uniformly loaded with force, *F,* until failure. The unconfined compressive strength, $R_{c}$ (N/mm^2^), was calculated as:

(3) $$R_{c}=\frac{F}{Ac}\;,$$

where F (N) is the maximum force until failure sustained by the specimen and Ac (mm^2^) is the cross-sectional area of the specimen.

#### 2.3.2. Indirect Tensile Strength ($R_{it}$)—Indirect Diametrical Tensile (IDT) Test

Indirect diametrical tensile (IDT) strength tests were conducted using a method based on EN-13286-42 [11] for cylindrical specimens with diameter *ϕ* = 100 mm and height *h* *=* 60 mm. The specimen was loaded monotonically with a vertical force, *F*, inducing horizontal tensile stress at a rate of less than 0.2 MPa/s until failure (monotonic load test). The indirect tensile strength, $R_{it}$ (N/mm^2^), was calculated as:

(4) $$R_{it}=\frac{2\cdot F}{\pi \cdot H\cdot \phi }\;$$

where F (N) is the maximum force to failure, *H* (mm) is the length of the specimen, and *ϕ* is the diameter of the specimen (mm).

#### 2.3.3. Dynamic Stiffness Modulus ($S_{m}$)—IDT Test

The dynamic stiffness modulus was tested by an indirect tensile method based on EN 12697-26 under a dynamic load, adjusting the method for the specific properties of hydraulically bound mixtures, such as brittleness and low deformability. Although this method was not designed to test hydraulically bound mixtures, its suitability has been demonstrated in previous studies [26,38]. The specimens were loaded cyclically with a repetitive force pulse of $F=30\%\cdot F_{max}$, where $F_{max}$ is the vertical destructive force. This induces a horizontal tensile strain in the range of 0.001–0.002. Generally, in determining the stiffness of hydraulically bound mixtures, the ultimate strengths of the material are assumed to be no more than 50% [39,40] and 30% [30]. The test-load level used in IDT stiffness testing was derived from the fragility of the material, and it should not be greater than 0.002 [38] to avoid fatigue effects and to maintain the deformation in the elastic range. A haversine-waveform load with a force-increase rate of 0.248 s and period of 3 s was used to study the stiffness modulus. The force-increase rate did not significantly affect the stiffness modulus values, as described by previous studies [26,38]; therefore, the standard frequency used in asphalt concrete testing of 0.33 Hz was applied for cement mixture testing. The stiffness–IDT dynamic modulus, $S_{m}$ (MPa), was determined as:

(5) $$S_{m}=\frac{F\cdot \left(\nu +0.27\right)}{\left(z\cdot h\right)},\;$$

where F (N) is the peak value of vertically applied force, z (mm) is the amplitude of horizontal deformation obtained during the force cycle, h (mm) is the average specimen length, and v is the Poisson ratio (v = 0.25). 

## 3. Results and Discussion

### 3.1. Unconfined Compressive Strength ($R_{c}$)

The average values of compressive strength for each mixture composition (i.e., compaction ratio, $D_{Pr}$, and cement addition) are presented in Table 3.

**Figure 5:**
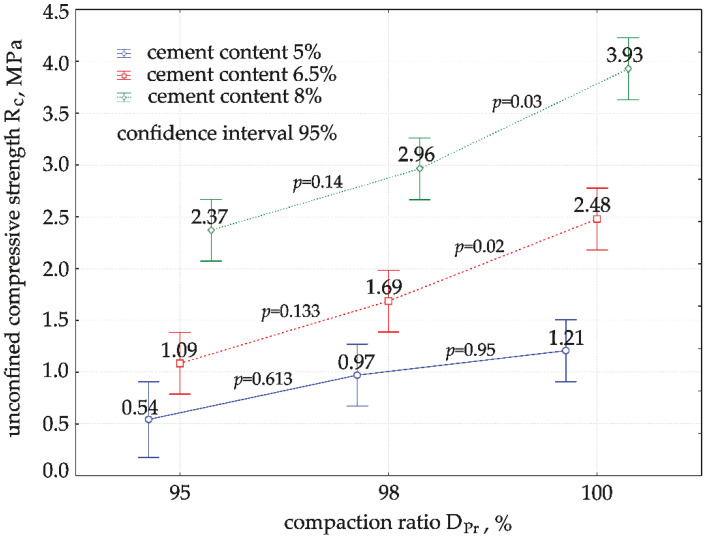
Diagram of variance of $R_{c}$ values and significance of differences between pairs of means.

The normality of the distribution of compressive strength test results and the homogeneity of variance in each of the nine groups were checked before the analysis using the Shapiro–Wilk and Levene’s tests. The distribution of the results was found to be normal with *W* ≥ 0.77 and *p*-value ≥ 0.051. The variance was found to be homogeneous at *p*-value = 0.61 > 0.05. Moreover, ANOVA was performed. The results are shown in Figure 5. 

**Figure 6:**
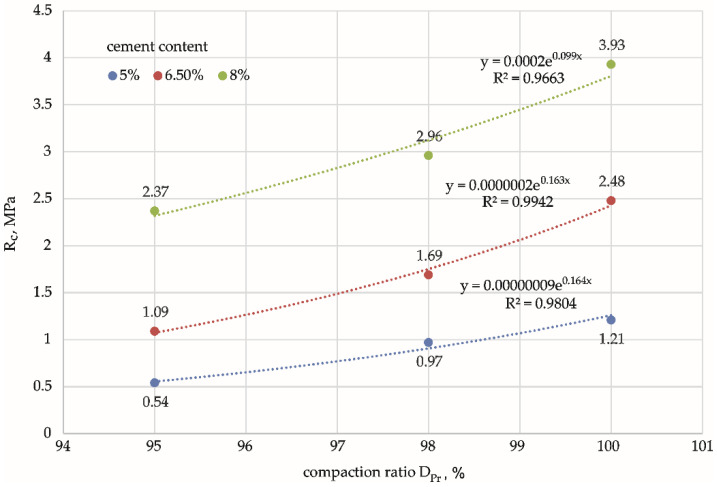
Effect of compaction ratio, $D_{Pr}$, on compressive strength, $R_{c}$, for mixtures with different cement contents. Cement content: **━** 5%, **━** 6.5%, **━** 8%.

One-dimensional (1D) tests of significance of variance for compressive strength, $R_{c}$, showed that both the compaction ratio and cement addition were variables that significantly affected the average compressive strength, with calculated significance values *p* < 0.05. However, no interaction was found between the factors ($D_{Pr}$*cement content) (*p* > 0.05): the effects of the compaction ratio and cement addition are independent of each other and should be considered separately. An analysis was therefore carried out by examining the effect of $D_{Pr}$ at the level of each cement addition. Tukey’s post hoc test was used to check the significance of differences between pairs of mean $R_{c}$ values in the groups. Calculations showed that, for $D_{Pr}$ = 95% to 98% compaction, the average compressive strength values depending on $D_{Pr}$ did not differ significantly (*p* > 0.05) for all the cement additions. However, in the compaction range $D_{Pr}$ = 98% to 100%, the difference in compressive strength values was significant (*p* < 0.05) for “harder” specimens with higher cement content (6.5% and 8%). For small cement additions (5%), the effect of the compaction ratio on compressive strength was insignificant over the entire range analysed, from 95% to 100%. Figure 6 shows the functional relationship between $R_{c}$ and $D_{Pr}$ for each cement addition.

The functions determined above indicate that an exponential relationship exists between the compaction ratio and the compressive strength, with a coefficient of determination *R*^2^ = 0.97–0.99 for all cement additions. It was observed that as the compaction ratio increased, compressive strength also increased. This increase was more pronounced for cement–soil specimens with a higher cement content (6.5% and 8%) than for specimens with lower strength (5%), as shown by ANOVA.

Specimens with higher compressive strengths were more sensitive to changes in compaction, especially in the upper limits of $D_{Pr}$ = 98–100%, than specimens with low binder content and low strength. 

### 3.2. Indirect Tensile Strength ($R_{it}$)—IDT Test

The results of the indirect tensile strength, $R_{it}$, tests are presented in Table 4.

**Figure 7:**
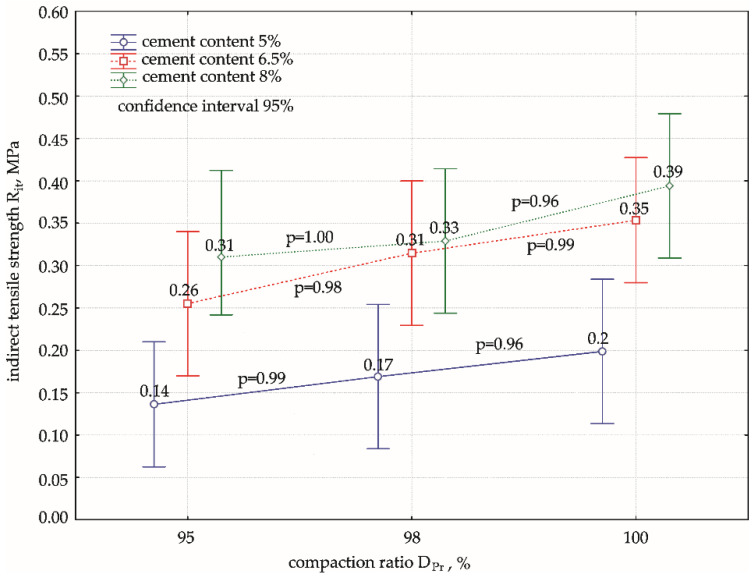
Diagram of variance of $R_{it}$ values and significance of differences between pairs of means. Cement content: **━** 5%, **━** 6.5%, **━** 8%.

After checking the normality of the distribution of the indirect tensile strength results, where *W* ≥ 0.81 and *p*-value ≥ 0.14 were obtained for the Shapiro–Wilk test, and finding the homogeneity of variance, *p*-value = 0.891 > 0.05 for the Levene’s test in each group, ANOVA was performed, and the results are shown in Figure 7.

One-dimensional tests of the significance of bivariate variance showed that the compaction ratio did not significantly affect the variation in mean indirect tensile strength, as confirmed by the calculated *p*-value = 0.081 > 0.05. However, the effect of the cement addition factor was significant (*p* < 0.05); thus, the effect of the compaction ratio at the level of each cement addition was investigated separately as in the analysis of compressive strength, $R_{c}$, results. The calculations showed no interaction between the factors ($D_{Pr}$*cement content) (*p* = 0.957 > 0.05): the effects of the compaction ratio and cement addition are independent of each other. The analysis of the test results presented in Figure 7 did not show any significant effect of the compaction ratio on the values of indirect tensile strength. The differences between the mean tensile strength values as a function of $D_{Pr}$ were not statistically significant for each cement addition. The insignificance of these differences is further confirmed by the overlap of the ranges of the 95% confidence intervals for the individual group of means over the entire range of densities analysed, $D_{Pr}$ = 95–100%.

**Figure 8:**
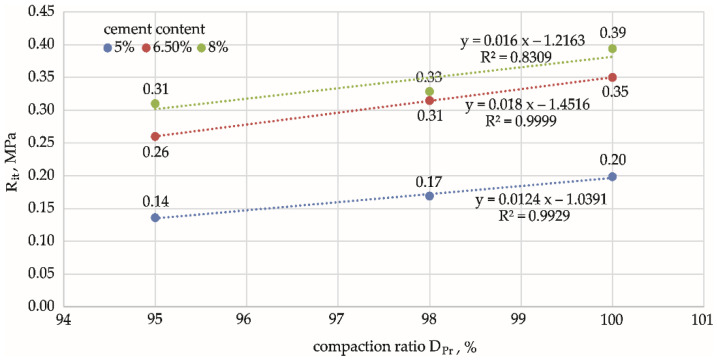
Effect of compaction ratio, $D_{Pr}$, on indirect tensile strength, $R_{it}$, for mixtures with different cement contents. Cement content: **━** 5%, **━** 6.5%, **━** 8%.

As shown in Figure 8, a linear relationship, $R_{it}=a\cdot D_{Pr}-b$, exists between the compaction ratio and the indirect tensile strength , with a coefficient of determination $R^{2}$ = 0.83–0.99 for all cement additions. It was observed that as the compaction ratio increased, there was an obvious increase in indirect tensile strength, the nature of which was approximately the same for different cement additions: the lines of dependence were parallel to each other. This was not found in the analysis of compressive strength. The rate of change in the increase in $R_{it}$ strength was low and mild, as evidenced by the non-significant differences between pairwise means (*p* > 0.05) at different $D_{Pr}$, as previously demonstrated by ANOVA.

### 3.3. Dynamic Stiffness Modulus ($S_{m}$)—IDT Test

The dynamic stiffness modulus test results are presented in Table 5.

**Figure 9:**
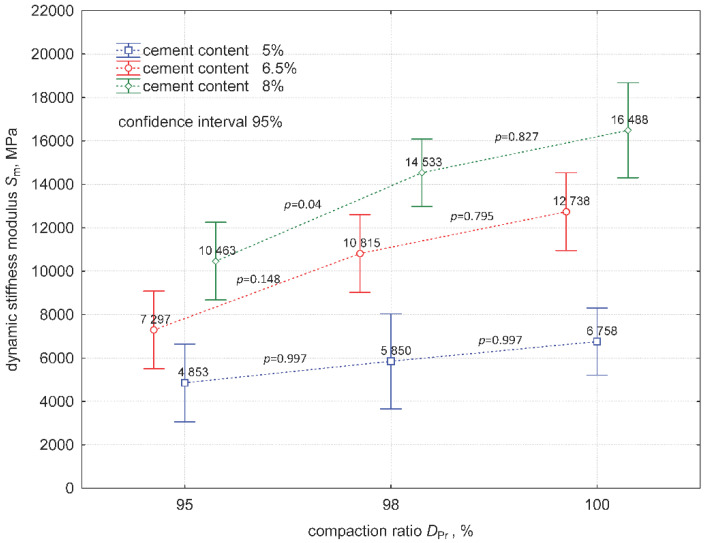
Diagram of variance of $S_{m}$ values and significance of differences between pairs of means.

ANOVA was performed after checking the normality of the distribution of the dynamic stiffness modulus results, where *W* ≥ 0.81 and *p*-value ≥ 0.139 were obtained for the Shapiro–Wilk test, and finding the homogeneity of variance, *p*-value = 0.242 > 0.05, using Levene’s test in each group. The results are shown in Figure 9.

Moreover, 1D tests of significance for the dynamic stiffness modulus showed that both the compaction ratio and cement addition were variables that significantly affected the average value, and this was confirmed by the calculated values of *p* < 0.05. The calculations showed no interaction between the factors ($D_{Pr}$*cement content) (*p* > 0.05), indicating that the effect of the compaction ratio and cement addition are independent of each other and should be considered separately. An analysis was then carried out by examining the effect of the compaction ratio on the dynamic stiffness modulus value at the level of each cement addition.

**Figure 10:**
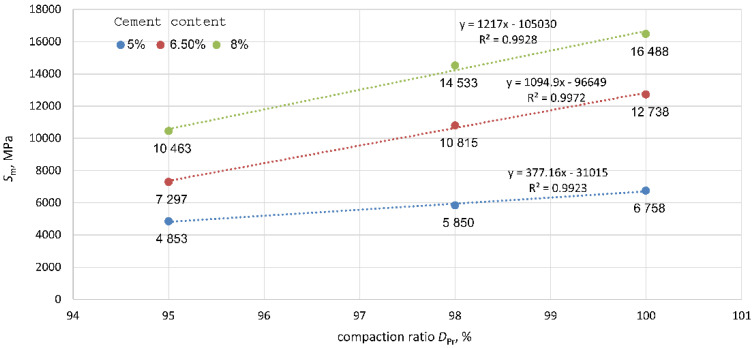
Influence of the compaction ratio, $D_{Pr}$, on the value of the dynamic stiffness modulus, $S_{m}$.

Tukey’s test, which examines the significance of differences between pairs of mean dynamic stiffness modulus values in the tested groups, did not show any significant effect of $D_{Pr}$ on dynamic stiffness modulus values, except for the pair of results for the mixture with 8% cement content in the range of $D_{Pr}$ = 95–98% (*p* = 0.04). On the other hand, significant differences in dynamic stiffness modulus, $S_{m}$, were apparent by comparing the $S_{m}$ values for mixtures with a higher cement content (6.5% and 8%) at extreme compaction levels of 95% and 100% (*p* = 0.06, *p* = 0.07). In addition, all other combinations of means within groups showed a lack of significance of the effect of $D_{Pr}$ on the value of the dynamic stiffness modulus. Figure 10 shows the functional relationship between $S_{m}$ and $D_{Pr}$ for each cement addition.

As shown in Figure 10, a linear relationship exists between the compaction ratio, $D_{Pr}$, and dynamic stiffness modulus $S_{m}=a\cdot D_{Pr}-b$, with a coefficient of determination $R^{2}$ = 0.99 for all cement additions. It is apparent that the value of the compaction ratio increased as the dynamic stiffness modulus increased. The rate of increase was approximately the same for cement additions of 6.5% and 8% (the relationship lines were parallel to each other). However, for the weakest specimens with the smallest cement addition (5%), the growth rate was the lowest and gentlest, and the effect of the compaction ratio on the dynamic stiffness modulus was less than that for larger cement additions. 

### 3.4. Analysis of Normalised $R_{c}$, $R_{it}$, and $S_{m}$

**Figure 11:**
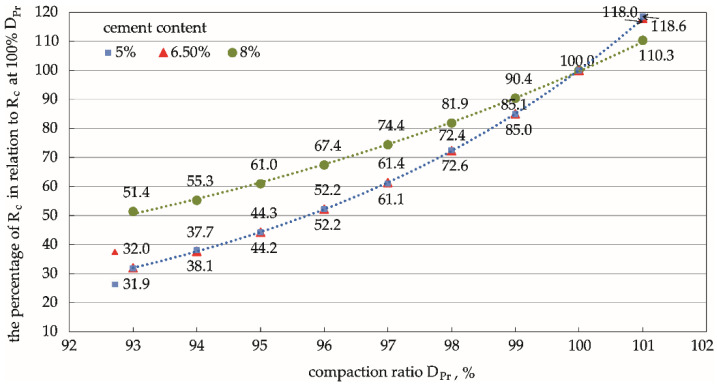
Corresponding relationships between the percentage of unconfined compressive strength, $R_{c}$, under different compaction degrees in relation to $R_{c}$ at 100% *D_Pr_*. Cement content: **━** 5%, **━** 6.5%, **━** 8%.

**Figure 12:**
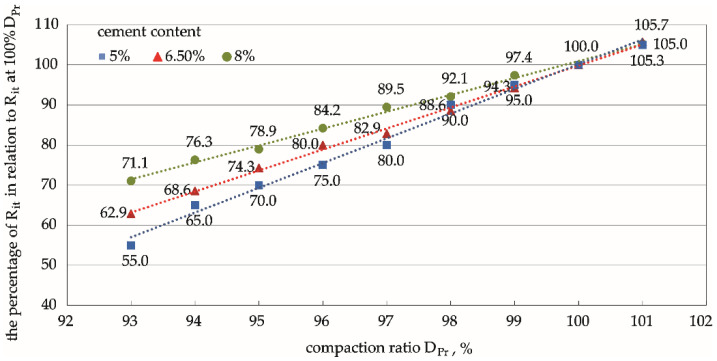
Corresponding relationships between the percentage of indirect tensile strength, $R_{it}$, under different compaction ratios in relation to $R_{it}$ at 100% *D_Pr_.* Cement content: **━** 5%, **━** 6.5%, **━** 8%.

**Figure 13:**
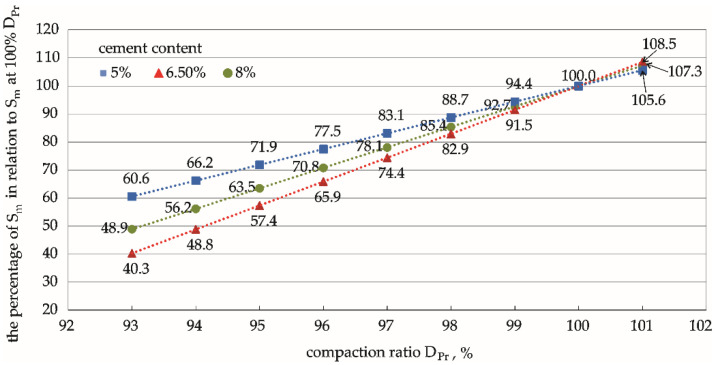
Corresponding relationships between the percentage of dynamic stiffness modulus, $S_{m}$, under different compaction ratios in relation to $S_{m}$ at 100% $D_{Pr}$. Cement content: **━** 5%, **━** 6.5%, **━** 8%.

The functional relationships between the compaction ratio and the various mechanical parameters (Figure 6, Figure 8 and Figure 10) were determined for various cement contents in the mixture using the results of compressive strength, indirect tensile strength, and dynamic stiffness modulus tests at three compaction levels ($D_{Pr}$ = 95%, 98%, and 100%). These results can be extrapolated to a wider compaction-level range through regression. The percentage values of the mechanical parameters $R_{c}$, $R_{it}$, and $S_{m}$ calculated in relation to the values of these parameters determined on specimens compacted at 100% $D_{Pr}$ illustrate the effect of the compaction ratio on the strength parameters of the cement-stabilised soil (Figure 11, Figure 12 and Figure 13). This comparison provides valuable practical information on the percentage by which the value of a given parameter changes with compaction, and the rate of this change.

The analysis showed that the unconfined compressive strength was the parameter most sensitive to a change in compaction ratio, as shown in Table 6 and Figure 11. The rate of these changes depended on the hardness of the specimen (i.e., on cement addition). The average rate of change in stronger specimens with a higher cement addition (8%) was much slower than the rate of change in weaker specimens containing 6.5% and 5% cement.

A similar relationship was observed when analysing the $R_{it}$ tensile strength, where stronger specimens with 8% cement content were less susceptible to changes in the compaction ratio than specimens containing less cement (5% and 6.5%). The decrease in indirect tensile strength as the compaction ratio decreased was slower than the decrease in compressive strength.

In conclusion, stronger specimens with a higher cement content were more resistant to changes in the compaction ratio than weaker specimens with a lower strength. In addition, the modulus showed a different trend than the percentage dependence of compressive strength and indirect tensile strength on the compaction ratio values discussed above. Note that there is no rule that stronger specimens are more resistant to changes in the parameter value with a change in the compaction ratio. The mixture with 5% cement content proved to be the most resistant to compaction ratio changes.

The compressive strength was the parameter most sensitive to compaction changes, as shown in Figure 11, Figure 12 and Figure 13. The differences between the pairs of means are apparent. The strength of the specimen increased with the cement content. On the other hand, the changes in compressive strength were not significant for weaker specimens, as is apparent for specimens with the lowest cement content (5%).

**Figure 14:**
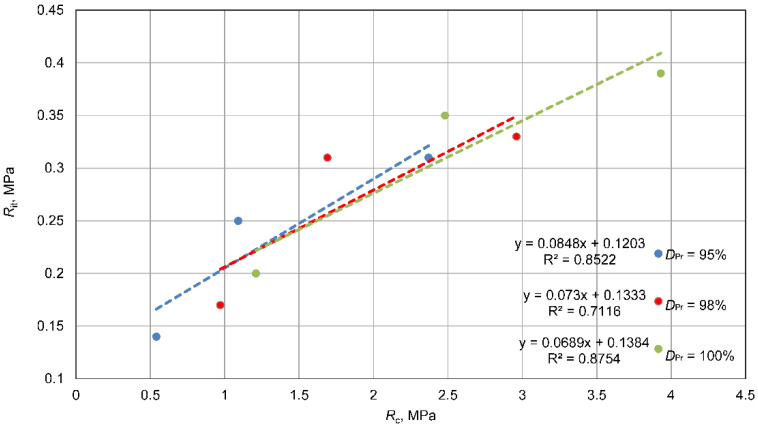
Relationships between unconfined compressive strength, $R_{c}$, and indirect tensile strength, *R_it_*.

**Figure 15:**
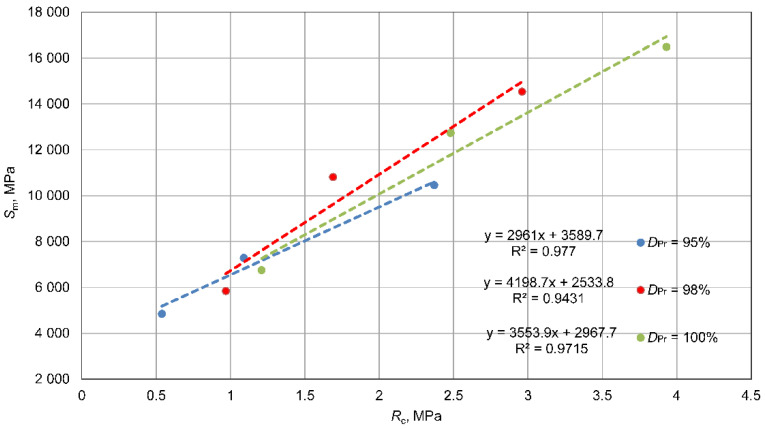
Relationships between unconfined compressive strength, $R_{c}$, and dynamic stiffness modulus, $S_{m}$.

**Figure 16:**
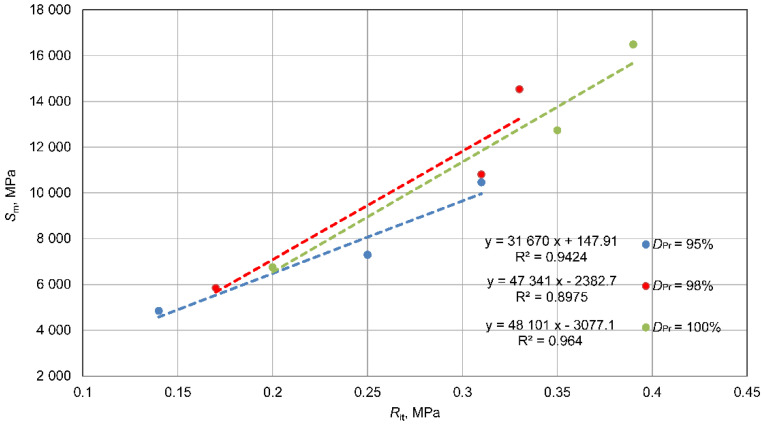
Relationships between indirect tensile strength, $R_{it}$, and dynamic stiffness modulus, $S_{m}$.

An analysis of the relationship between the tested parameters, compressive strength, indirect tensile strength, and dynamic stiffness modulus, was carried out separately for each density state, *D_Pr_* = 0.95%, 98%, and 100%. The results of these analyses are presented in Figure 14, Figure 15 and Figure 16.

There are linear correlations between the studied parameters for each compaction condition. Regardless of the *D_Pr_* values, the relationships are very similar to each other. This was expected, since the parameters were compared with each other in the same state of compaction. For the relationship between *R_it_* and *R_c_* strength, very similar linear relationships were observed, with a coefficient of determination *R*^2^ = 0.71–0.88, confirming that indirect tensile strength, *R_it_*, accounts for 10–20% of *R_c_*, which confirms known dependencies. The relationships between *S_m_* modulus and *R_c_* or *R_it_* strengths with a coefficient of determination *R*^2^ = 90 – 98 were very similar to each other, which should be related to the good correlation between *R_c_* and *R_it_* strength. It should be noted that the dependences for mixtures with density *D_Pr_* = 98% and 100% were very similar to each other, and the mixture with *D_Pr_* = 95% stood out a little from the others. This is related to the relationship shown in Figure 14, and it is a difference within the error range caused by the scattering of results. In conclusion, it should be stated that the relationships between tested parameters were similar for each compaction condition in the tested range.

## 4. Conclusions

The analysis of the test results of soil stabilisation with low-strength cement showed that the values of the mechanical parameters: unconfined compressive strength, $R_{c}$, indirect tensile strength, $R_{it}$, and dynamic stiffness modulus, $S_{m},\;$ depend on the state of compaction. For the compressive strength,  this dependence was exponential in form, whereas for the indirect tensile strength and dynamic stiffness modulus, it was linear. The compaction ratio in the upper range, $D_{Pr}$ = 98–100%, had a significant influence on the unconfined compressive strength. On the other hand, the changes in compressive strength were much smaller and not statistically significant when $D_{Pr}$ < 98%. Moreover, the influence of the compaction ratio on indirect tensile strength and the dynamic stiffness modulus was not as clear and statistically significant as in the case of compressive strength. 

It was observed that the rates of change in compressive strength and indirect tensile strength depend on the hardness of the specimen, which in turn depends on the cement addition. The comparisons of the percentage changes in compressive strength and indirect tensile strength at different compaction ratios with these parameters at the standard compaction of $D_{Pr}$ = 100% showed that the strongest mixtures were the least resistant to changes in the compaction ratio. Moreover, weaker mixtures with a lower cement content were found to be less resistant to compaction ratio changes for compressive strength and indirect tensile strength. A different trend was observed when the dynamic stiffness modulus values were analysed at different compaction states: the mixture with the lowest cement content was the least susceptible to compaction ratio changes.

The values of the parameters at a compaction equal to 100% $D_{Pr}$ were taken as the standards for comparison in the analyses. The percentage values of the parameters relative to these standard levels (Figure 11, Figure 12 and Figure 13 and Table 6) have great engineering utility because they can be estimated at any compaction level, even when it is not technologically possible to obtain satisfactory in situ compaction. Furthermore, they can be used to predict how weak the strength parameter will be in relation to the assumptions made at 100% $D_{Pr}$.

There were linear correlations between the tested parameters: indirect tensile strength, *R*_it_, unconfined compressive strength,$\;R_{c}$, and dynamic stiffness modulus, $S_{m}$, for each compaction condition (Figure 14, Figure 15 and Figure 16). It should be stated that the relationships between these tested parameters (*R_it_*$,\;R_{c}$,$\;S_{m}$) were constant for each compaction state.

## Figures and Tables

**Table 1:** Energy used to obtain the assumed compaction ratio of the specimens.

Number of 2.5 kg Hammer Blows per Specimen with *ϕ* = 100 and *h* = 120 mm	$\%\;D_{Pr}$	E (J/cm^3^)	E (%)
15	95	0.1191	20
36	98	0.2859	48
75	100	0.5955	100

$\%\;D_{Pr}$: compaction ratio; E: compacting energy.

**Table 2:** Properties of the soil–cement mixtures.

Mixture	Max Dry Density, $\rho_{ds}$ (g/cm^3^)	Optimum Moisture Content, OMC (%)
100% sand	1.833	8.2
95% sand + 5% cement	1.897	8.5
93.5% sand + 6.5% cement	1.903	9.5
92% sand + 8% cement	1.921	9.5

**Table 3:** Test results: unconfined compressive strength, $R_{c}$, under different compaction ratios, $D_{Pr}$, and various cement dosages.

Cement Dosage (%)	$\;D_{Pr}$ (%)	Average $\;R_{c}\;$ (MPa)	Standard Deviation (SD)
5	95	0.54	0.067
	98	0.97	0.033
	100	1.21	0.055
6.5	95	1.09	0.076
	98	1.69	0.255
	100	2.48	0.141
8	95	2.37	0.387
	98	2.96	0.426
	100	3.93	0.287

**Table 4:** Test results: indirect tensile strength, $R_{it}$, under different compaction ratios and cement dosages.

Cement Dosage (%)	Compaction Ratio, $D_{Pr}$ (%)	Average Indirect Tensile Strength, *R_it_* (MPa)	Standard Deviation (SD)
5	95	0.14	0.047
	98	0.17	0.048
	100	0.20	0.054
6.5	95	0.25	0.059
	98	0.31	0.105
	100	0.34	0.089
8	95	0.31	0.034
	98	0.33	0.104
	100	0.39	0.024

**Table 5:** Test results: dynamic stiffness modulus under different compaction ratios, $D_{Pr}$, and cement dosages.

Cement Content (%)	Compaction Ratio, $D_{Pr}$ (%)	Dynamic Stiffness Modulus, $S_{m}$ (MPa)	Standard Deviation (SD)
5	95	4853	140.7
	98	5850	54.1
	100	6758	588.8
6.5	95	7297	1126.4
	98	10,815	323.8
	100	12,738	2265.9
8	95	10,463	1659.6
	98	14,533	1649.1
	100	16,488	3375.7

**Table 6:** Percentage values of the parameters under different compaction ratios in relation to values at 100% $D_{Pr}$.

Feature	$93\%\;D_{Pr}$	$95\%\;D_{Pr}$	$98\%\;D_{Pr}$	$100\%\;D_{Pr}$
% $R_{c}$	32–51	44–61	72–82	100
% $R_{it}$	55–71	70–79	89–92	100
% $S_{m}$	40–61	57–72	83–89	100

## Data Availability

The data that support the findings of this study are available from the corresponding author, C.K., upon reasonable request.
